# Avian Influenza and Ban on Overnight Poultry Storage in Live Poultry Markets, Hong Kong

**DOI:** 10.3201/eid1808.111879

**Published:** 2012-08

**Authors:** Y.H. Connie Leung, Eric H.Y. Lau, Li Juan Zhang, Yi Guan, Benjamin J. Cowling, J.S. Malik Peiris

**Affiliations:** Li Ka Shing Faculty of Medicine School of Public Health, The University of Hong Kong, Hong Kong Special Administrative Region, People’s Republic of China (Y.H.C. Leung, E.H.Y. Lau, B.J. Cowling, J.S.M. Peiris);; and State Key Laboratory of Emerging Infectious Diseases, The University of Hong Kong, Hong Kong (L.J. Zhang, Y. Guan)

**Keywords:** avian influenza, H9N2, poultry, live poultry markets, interventions, epidemiology, control, influenza, chickens, Hong Kong, H5N1

## Abstract

We analyzed ≈12 years of surveillance data on avian influenza in Hong Kong live poultry markets. A ban on keeping live poultry overnight in these markets reduced virus isolation rates by 84% in chickens (p = 0.006) and 100% (p = 0.01) in minor poultry.

Previous influenza pandemics originated from influenza viruses of birds (*1*). Live poultry markets play a crucial role in maintenance, amplification, and dissemination of avian influenza viruses (*2**,**3*) and are high-risk locations for potential zoonotic transmission of highly pathogenic avian influenza (HPAI) virus (H5N1) to humans (*4**,**5*). From September 1999 through May 2011, fecal dropping samples were collected monthly under the poultry cages in live poultry markets in Hong Kong as part of a systematic longitudinal avian influenza surveillance program. During the 12-year period of surveillance, several interventions were implemented by the Hong Kong government in response to outbreaks of influenza virus (H5N1) in live poultry markets and on poultry farms. In July 2001, a monthly rest day was first implemented; under this system, all poultry in live poultry markets must be sold or slaughtered at the end of the day, poultry stalls must be cleaned and disinfected, and the stalls must be left free of live poultry for 1 day before restocking any live poultry the next day. In February 2002, a ban on sales of live quail was implemented in because an influenza virus (H9N2) lineage commonly isolated from quail possessed the internal genes of the virus that caused the avian influenza (H5N1) outbreak in Hong Kong in 1997 (*6*). In response to further incursions of avian influenza (H5N1) into poultry markets and farms in Hong Kong, a second monthly rest day in live poultry markets was introduced in March 2003, and a complete ban on holding live poultry overnight in live poultry markets was implemented in July 2008.

Previously, we analyzed data from September 1999 through December 2005 and demonstrated that 1 rest day per month significantly reduced isolation rates of influenza virus in minor poultry (i.e., silkie chickens, pigeons, chukars, guinea fowls, and pheasants) but that an additional rest day each month did not significantly reduce the isolation rate further (*7*). In this follow-up study, which includes an additional 6 years of data, we investigated the effect of a ban on keeping live poultry overnight at live poultry markets on isolation rates of influenza A virus (H9N2) from chickens and minor poultry.

## The Study

When the live poultry market surveillance program began in September 1999, eight of a total of 80 live poultry markets were selected to represent the 3 major regions of Hong Kong: Hong Kong island, Kowloon, and the New Territories. Since then, the number of markets has declined, and by May 2011, only 5 of the 8 selected live poultry markets continued in operation (of a total of 39 operating live poultry markets). A total of 53,541 samples were collected during these 141 months of consecutive sampling.

We previously published data on the effect of introducing various interventions in live poultry markets, which included the ban on the sales of live quail and the introduction of rest days (*7*). In addition to collecting fecal droppings from the cage floors for virus isolation, we collected data on the total sales of chickens and minor poultry, the proportion of chickens imported as a ratio of the whole, the temperature and relative humidity, and the type of ventilation used, as described (*7*). Laboratory processing of the specimens was conducted as described (*7*). Samples collected in virus transport medium were inoculated into 9–11-day-old embryonated eggs, and allantoic fluid with positive hemagglutination was confirmed and subtyped using standard antiserum.

Because HPAI A virus (H5N1) is rarely detected in live poultry markets in Hong Kong, we used isolation rates of influenza A virus (H9N2) as an indicator of the effect of these interventions on avian influenza virus circulation. The median numbers of samples collected weekly from chickens and minor poultry were 107 (range 3–722) and 23 (range 1–397), respectively (see Technical Appendix for weekly numbers of samples). The Poisson generalized model (*8*) with influenza virus (H9N2) weekly isolation counts as the outcome variable was fitted as described (*7*) and adjusted for proportion of chickens imported; total sales of chickens and minor poultry; ventilation system; weekly average temperature; relative humidity; seasonal variations; sample size; and periods corresponding to the respective interventions: period I (no rest day), II (1 monthly rest day with quail being sold in the live poultry market), III (1 monthly rest day with elimination of live quail from the live poultry market), IV (2 monthly rest days), and V (ban on holding poultry overnight in live poultry market). These variables were considered potentially important confounders related to transmission efficiency of avian influenza virus (*9*), source, type, and volume of poultry.

Weekly virus isolation counts were analyzed from September 22, 1999, through May 31, 2011. A separate model for poultry and minor poultry was fitted, and all analyses were implemented by using R version 2.12.1 software (R Development Core Team, Vienna, Austria).

The Figure shows overall isolation rates by week for chicken and minor poultry from 1999 through 2011; the Table gives the parameter estimates for the final fitted models, which were adjusted for the effect of covariables that could affect the isolation of influenza in the study. For chickens and minor poultry, compared with the reference category of 2 monthly rest days, the ban on keeping live poultry overnight in live poultry markets was associated with dramatic and significant reduction of influenza virus (H9N2) isolation. The isolation rate of influenza virus (H9N2) among chickens declined 84% (adjusted relative risk 0.16; p = 0.006), and no influenza subtype H9N2 viruses were isolated from minor poultry after the ban on holding poultry overnight in live poultry markets was implemented. Higher volume of minor poultry sales was also significantly associated with higher isolation rate of influenza virus (H9N2).

**Figure:**
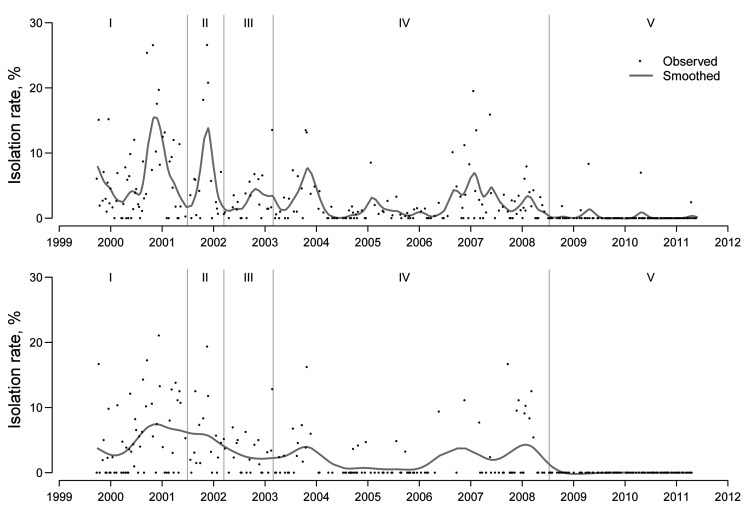
Weekly influenza virus A (H9N2) isolation rates for chickens (A) and minor poultry (B) in live poultry markets, Hong Kong, September 1999–May 2011. Vertical lines denote periods for different interventions: I, no rest day; II, 1 monthly rest day with quail sold in live poultry markets; III, 1 monthly rest day with no sales of quail in live poultry markets; IV,: 2 monthly rest days; V, ban on keeping live poultry overnight in live poultry markets.

**Table:** Poisson generalized linear models for influenza virus (H9N2) isolation rates in live poultry markets, by poultry type, Hong Kong, September 1999–May 2011*

Variable	Chickens			Minor poultry	
	aRR (95% CI)	p value		aRR (95% CI)	p value
Period					
No rest day	1.69 (0.91–3.15)	0.10		2.47 (1.23–4.98)	0.01
1 rest day with quail†	1.25 (0.73–2.15)	0.42		0.99 (0.49–2.01)	0.97
1 rest day without quail†	1.00 (0.60–1.64)	0.97		0.99 (0.53–1.85)	0.97
2 rest days	Reference			Reference	
Ban on keeping live poultry overnight in live poultry markets	0.16 (0.04–0.60)	0.006		‡	0.01‡
Proportion of chickens imported, per 10% increase	0.87 (0.73–1.02)	0.09		1.02 (0.79–1.32)	0.87
Total sales					
Chickens, per 100,000 sold	1.04 (0.98–1.09)	0.19		1.05 (0.98–1.13)	0.15
Minor poultry, per 100,000 sold	2.52 (1.49–4.25)	0.001		3.15 (1.54–6.44)	0.002
Chicken × minor poultry§	0.98 (0.97–1.00)	0.03		0.97 (0.95–0.99)	0.007
Ventilation system					
Natural ventilation	Reference			Reference	
Market economic air treatment system	1.02 (0.79–1.31)	0.89		1.02 (0.78–1.34)	0.87
Air conditioned	0.71 (0.42–1.22)	0.21		0.97 (0.56–1.68)	0.90
Temperature, °C	0.98 (0.99–1.02)	0.96		1.05 (0.96–1.16)	0.29
Relative humidity, %	1.00 (0.99–1.02)	0.63		0.99 (0.97–1.00)	0.10
Seasonality term¶					
α (cosine component)	0.19 (–0.19 to 0.58)	0.33		–0.10 (–0.57 to 0.37)	0.68
β (sine component)	0.30 (–0.11 to 0.70)	0.15		0.47 (–0.06 to 0.99)	0.08

*aRR, adjusted relative risk. †Indicates before and after ban on sales of live quail. ‡Reliable confidence interval cannot be estimated because of zero isolation of influenza virus (H9N2) from minor poultry after introduction of a ban on keeping live poultry overnight in live poultry markets. p value was calculated using likelihood ratio test. §Interaction term. ¶The seasonality coefficients α and β contribute to the estimated isolation rate in week *t* via the terms αcos(2π*t*/52) + βsin(2π*t*/52).

## Conclusions

A previous study that used a stochastic metapopulation model showed that frequent rest days in live poultry markets were effective for reducing transmission of avian influenza (H5N1) (*10*). Our findings show a large additional decline in the influenza virus (H9N2) isolation rate after implementation of a ban on keeping live poultry overnight, which suggests that this intervention has an even greater effect on reducing viral load in live poultry markets than the previous intervention of 1 or 2 rest days per month. While low pathogenic influenza virus (H9N2) was the indicator virus in our study, it is likely that these interventions would have comparable effects on highly pathogenic viruses such as avian influenza (H5N1); this effect has been demonstrated by mathematical modeling (*10*). Studies by others on social network analysis have shown that daily cage cleaning and disinfection of live poultry markets in southern China (*11*), and protective factors including removal of waste in Indonesia (*12*) contributed to a reduction of HPAI (H5N1) in live poultry markets. Taken together, these studies show that eliminating the carryover of live poultry in markets from one day to the next, in the form of rest days or a total ban, is highly effective for reducing viral amplification and persistence in live poultry markets and consequently minimizes zoonotic risk.

Technical Appendix Weekly number of samples and influenza virus (H9N2) isolation rates for live poultry markets, by poultry type, Hong Kong, September 1999–013;May 2011.

## References

[1] Shortridge KF. Pandemic influenza: a zoonosis? Semin Respir Infect. 1992;7:11–25. 1609163

[2] Kung NY, Morris RS, Perkins NR, Sims LD, Ellis TM, Bissett L, Risk for infection with highly pathogenic influenza A virus (H5N1) in chickens, Hong Kong, 2002. Emerg Infect Dis. 2007;13:412–8. 10.3201/eid1303.060365 17552094 PMC2725907

[3] Santhia K, Ramy A, Jayanisngsih P, Samaan G, Putra A, Dibia N, Avian influenza A H5N1 infections in Bali Province, Indonesia: a behavioral, virological and seroepidemiological study. Influenza Other Respi Viruses. 2009;3:81–9. 10.1111/j.1750-2659.2009.00069.x 19459276 PMC4634692

[4] Shortridge KF, Gao P, Guan Y, Ito T, Kawaoka Y, Markwell D, Interspecies transmission of influenza viruses: H5N1 virus and a Hong Kong SAR perspective. Vet Microbiol. 2000;74:141–7. 10.1016/S0378-1135(00)00174-7 10799786

[5] Mounts AW, Kwong H, Izurieta HS, Ho Y, Au T, Lee M, Case–control study of risk factors for avian influenza A (H5N1) disease, Hong Kong, 1997. J Infect Dis. 1999;180:505–8. 10.1086/314903 10395870

[6] Guan Y, Shortridge KF, Krauss S, Chin PS, Dyrting KC, Ellis TM, H9N2 influenza viruses possessing H5N1-like internal genomes continue to circulate in poultry in southeastern China. J Virol. 2000;74:9372–80. 10.1128/JVI.74.20.9372-9380.2000 11000205 PMC112365

[7] Lau EH, Leung YH, Zhang LJ, Cowling BJ, Mak SP, Guan Y, Effect of interventions on influenza A (H9N2) isolation in Hong Kong's live poultry markets, 1999–2005. Emerg Infect Dis. 2007;13:1340–7. 10.3201/eid1309.061549 18252105

[8] Serfling RE. Methods for current statistical analysis of excess pneumonia influenza deaths. Public Health Rep. 1963;78:494–506. 10.2307/4591848 19316455 PMC1915276

[9] Lowen AC, Mubareka S, Steel J, Palese P. Influenza virus transmission is dependent on relative humidity and temperature. PLoS Pathog. 2007;3:1470–6. 10.1371/journal.ppat.0030151 17953482 PMC2034399

[10] Fournié G, Guitian FJ, Mangtani P, Ghani AC. Impact of the implementation of rest days in live bird markets on the dynamics of H5N1 highly pathogenic avian influenza. J R Soc Interface. 2011;8:1079–89. 10.1098/rsif.2010.0510 21131332 PMC3119874

[11] Martin V, Zhou X, Marshall E, Jia B, Fusheng G, Francodixon MA, Risk-based surveillance for avian influenza control along poultry market chains in South China: the value of social network analysis. Prev Vet Med. 2011;102:196–205. 10.1016/j.prevetmed.2011.07.007 21925753 PMC7127115

[12] Indriani R, Samaan G, Gultom A, Loth L, Indryani S, Adjid R, Environmental sampling for avian influenza virus A (H5N1) in live-bird markets, Indonesia. Emerg Infect Dis. 2010;16:1889–95. 21122218 10.3201/eid1612.100402 PMC3294595

