# Supplementary material for: Avian Influenza and Ban on Overnight Poultry Storage in Live Poultry Markets, Hong Kong
Source: Emerg Infect Dis. 2012 Aug;18(8):1339–41. doi: 10.3201/eid1808.111879 (PMC3414033; doi:10.3201/eid1808.111879)
Supplement: Technical Appendix — Weekly number of samples and influenza virus (H9N2) isolation rates for live poultry markets, by poultry type, Hong Kong, September 1999–013;May 2011. [file 11-1879_Techapp-s1.pdf]

Article DOI: http://dx.doi.org/10.3201/eid1808.111879

Avian Influenza and Ban on Overnight Storage in Live Poultry Markets, Hong Kong

Technical Appendix

Technical Appendix Table. Weekly number of samples and influenza virus (H9N2) isolation rates for live poultry markets, by poultry type, Hong Kong, September 1999–May 2011

		Chickens		Minor Poultry	
Year/Interventions	Week	No. samples	Isolation %	No. samples	Isolation %
1999/no intervention	38	33	6.1	28	0.0
	40	53	15.1	6	16.7
	41	104	1.9	23	0.0
	44	112	2.7	52	1.9
	45	113	7.1	20	5.0
	47	101	3.0	10	0.0
	48	100	1.0	49	0.0
	49	147	5.4	44	2.3
	50	191	15.2	51	9.8
	51	44	2.3	23	0.0
	52	44	4.5	13	0.0
2000	2	242	1.7	84	2.4
	4	81	0.0	29	0.0
	6	114	2.6	32	0.0
	7	159	6.9	87	10.3
	11	93	0.0	25	0.0
	12	66	0.0	21	4.8
	13	46	2.2	16	0.0
	15	77	7.8	34	0.0
	16	93	0.0	26	3.8
	17	51	5.9	27	0.0
	18	3	0.0	8	0.0
	19	62	6.5	28	3.6
	20	61	9.8	33	12.1
	21	24	0.0	31	3.2
	23	144	1.4	23	4.3
	24	183	12.0	104	1.0
	25	155	3.9	134	8.2
	26	157	4.5	107	6.5
	28	94	2.1	33	0.0
	29	58	1.7	36	5.6
	30	24	0.0	25	4.0
	32	91	1.1	32	6.3
	33	46	8.7	35	14.3
	34	33	3.0	21	0.0
	36	54	3.7	59	10.2
	37	67	25.4	58	17.2
	42	203	7.4	104	10.6
	43	64	26.6	18	5.6
	46	137	10.2	31	0.0
	47	131	17.6	67	7.5
	49	127	19.7	38	21.1
	50	122	8.2	98	13.3
2001	1	216	12.5	102	3.9
	3	38	13.2	17	0.0
	5	116	0.0	40	0.0
	7	144	0.0	29	0.0
	8	23	8.7	25	8.0
	10	128	9.4	47	12.8
	11	107	4.7	33	3.0

Year/Interventions	Week	Chickens		Minor Poultry	
		No. samples	Isolation %	No. samples	Isolation %
	12	25	12.0	7	0.0
	14	226	1.8	29	13.8
	16	115	0.0	27	11.1
	17	34	0.0	11	0.0
	18	88	11.4	24	12.5
	19	56	1.8	28	10.7
	24	396	0.3	397	5.3
1 monthly rest day	29	454	3.5	152	2.0
	30	360	0.0	184	0.0
	33	333	6.0	195	3.1
	34	34	5.9	8	12.5
	35	424	0.5	270	1.5
	38	359	4.2	232	7.3
	39	324	0.0	136	1.5
	42	347	18.2	228	8.3
	46	158	26.6	62	19.4
	47	178	20.8	170	11.8
	50	245	5.7	188	3.2
	51	89	0.0	68	0.0
2002	3	326	2.5	176	5.7
	4	68	1.5	30	0.0
	5	297	0.7	129	2.3
	8	338	7.1	153	4.6
Ban of live quail sales	11	162	0.6	58	5.2
	12	225	0.9	81	3.7
	16	414	0.0	151	0.0
	20	240	1.7	72	6.9
	21	171	3.5	43	2.3
	24	151	1.3	44	4.5
	25	202	2.5	80	5.0
	28	144	0.0	32	0.0
	29	193	0.0	72	0.0
	33	396	3.8	96	6.3
	34	113	0.0	38	0.0
	37	125	5.6	50	2.0
	38	202	3.5	48	0.0
	42	385	6.8	117	4.3
	46	117	6.0	40	5.0
	47	192	2.1	78	1.3
	49	95	0.0	31	0.0
	50	244	6.6	77	0.0
2003	3	139	1.4	32	3.1
	4	207	1.4	77	0.0
	7	180	1.7	59	3.4
2 monthly rest days	8	133	13.5	39	12.8
	11	123	0.0	35	0.0
	12	169	0.6	66	0.0
	15	95	0.0	42	2.4
	16	202	1.0	68	0.0
	20	221	3.2	77	2.6
	21	152	0.0	34	0.0
	24	93	0.0	16	0.0
	25	234	3.0	69	0.0
	29	245	7.3	74	6.8
	30	99	1.0	14	0.0
	33	100	1.0	39	2.6
	34	233	6.4	66	4.5
	38	395	4.6	96	7.3
	39	194	0.0	59	1.7
	42	126	13.5	26	3.8
	43	228	13.2	37	16.2
	47	348	3.7	67	6.0
	51	350	4.9	0	–
2004	2	349	1.4	41	0.0
	3	145	4.1	27	0.0
	7	231	0.9	0	–
	11	75	0.0	0	–
	12	156	0.0	8	0.0
	15	343	1.7	0	–

Year/Interventions	Week	Chickens		Minor Poultry	
		No. samples	Isolation %	No. samples	Isolation %
	16	441	0.0	10	0.0
	18	124	0.0	0	–
	20	399	0.0	0	–
	21	140	0.0	0	–
	27	254	0.0	69	0.0
	29	338	0.0	72	0.0
	30	122	0.0	38	0.0
	33	336	1.2	62	0.0
	34	104	0.0	21	0.0
	35	103	0.0	22	0.0
	36	168	1.8	49	0.0
	37	354	0.8	55	3.6
	38	85	0.0	21	0.0
	40	177	0.6	42	0.0
	41	116	0.0	19	0.0
	42	208	1.0	48	4.2
	46	338	1.2	50	0.0
	49	100	0.0	34	0.0
	50	4	0.0	64	4.7
2005	3	352	8.5	36	0.0
	7	299	2.0	68	0.0
	11	309	0.6	45	0.0
	15	88	1.1	6	0.0
	16	211	0.9	39	0.0
	20	417	2.6	34	0.0
	24	89	0.0	6	0.0
	25	231	0.4	29	0.0
	29	456	3.3	62	4.8
	33	185	0.5	23	0.0
	34	111	0.0	1	0.0
	38	325	0.3	31	3.2
	40	128	0.8	8	0.0
	41	121	0.0	13	0.0
	42	295	0.3	33	0.0
	43	112	0.0	9	0.0
	46	78	0.0	8	0.0
	47	149	1.3	4	0.0
	50	262	1.5	9	0.0
2006	2	286	1.0	26	0.0
	6	136	1.5	14	0.0
	8	407	0.0	42	0.0
	9	98	0.0	8	0.0
	10	98	0.0	0	–
	12	278	0.0	0	–
	16	297	0.3	17	0.0
	20	722	2.4	32	9.4
	24	206	0.0	0	–
	25	108	0.0	0	–
	29	314	0.3	0	–
	34	326	10.1	0	–
	38	288	4.9	12	0.0
	42	304	3.3	0	–
	43	121	0.0	0	–
	44	127	1.6	0	–
	45	145	0.0	0	–
	46	241	11.2	9	11.1
	47	56	3.6	0	–
	50	10	0.0	0	–
	51	254	8.3	0	–
2007	3	287	19.5	0	–
	4	117	0.0	0	–
	5	24	4.2	0	–
	6	356	13.5	0	–
	9	107	2.8	13	7.7
	10	114	0.0	8	0.0
	12	239	2.1	2	0.0
	13	99	0.0	0	–
	16	229	0.9	7	0.0
	20	107	15.9	42	2.4

Year/Interventions	Week	Chickens No. samples	Chickens Isolation %	Minor Poultry No. samples	Minor Poultry Isolation %
	21	104	3.8	2	0.0
	25	196	0.0	28	0.0
	29	173	1.2	30	0.0
	33	115	1.7	10	0.0
	34	167	3.6	13	0.0
	38	297	1.0	6	16.7
	40	107	0.0	21	0.0
	41	124	0.0	18	0.0
	42	293	2.7	38	0.0
	43	126	0.0	9	0.0
	45	116	2.6	5	0.0
	47	156	0.6	21	9.5
	49	87	3.4	9	11.1
	51	164	1.2	21	0.0
2008	1	108	0.0	11	0.0
	2	126	1.6	7	0.0
	3	215	6.0	44	9.1
	4	172	1.2	39	10.3
	5	126	7.9	0	–
	8	150	4.0	36	8.3
	10	164	1.8	8	12.5
	12	164	4.3	37	5.4
	14	62	0.0	0	–
	15	75	0.0	0	–
	16	99	0.0	25	0.0
	17	63	0.0	12	0.0
	19	124	3.2	0	–
	21	150	2.0	31	0.0
	23	594	1.0	50	0.0
Ban on holding live poultry overnight in live poultry markets	27	63	0.0	0	–
	28	90	0.0	1	0.0
	29	53	0.0	3	0.0
	30	139	0.0	1	0.0
	31	16	0.0	2	0.0
	36	94	0.0	5	0.0
	38	153	0.0	15	0.0
	39	53	0.0	7	0.0
	41	54	1.9	8	0.0
	42	87	0.0	27	0.0
	44	20	0.0	4	0.0
	45	53	0.0	13	0.0
	46	42	0.0	18	0.0
	48	21	0.0	6	0.0
	53	107	0.0	13	0.0
2009	3	13	0.0	0	–
	6	14	0.0	5	0.0
	7	82	1.2	29	0.0
	8	14	0.0	12	0.0
	11	31	0.0	0	–
	12	250	0.0	71	0.0
	14	23	0.0	5	0.0
	15	60	8.3	4	0.0
	16	89	0.0	14	0.0
	19	105	0.0	20	0.0
	20	43	0.0	7	0.0
	24	50	0.0	11	0.0
	25	90	0.0	7	0.0
	28	47	0.0	19	0.0
	29	125	0.0	21	0.0
	32	11	0.0	2	0.0
	33	59	0.0	9	0.0
	34	33	0.0	13	0.0
	35	15	0.0	1	0.0
	36	19	0.0	6	0.0
	37	104	0.0	17	0.0
	40	14	0.0	3	0.0
	41	25	0.0	3	0.0
	42	39	0.0	2	0.0

Year/Interventions	Week	Chickens No. samples	Chickens Isolation %	Minor Poultry No. samples	Minor Poultry Isolation %
	43	39	0.0	4	0.0
	44	19	0.0	2	0.0
	45	11	0.0	6	0.0
	46	76	0.0	11	0.0
	48	42	0.0	2	0.0
	49	80	0.0	0	–
2010	1	46	0.0	3	0.0
	2	30	0.0	4	0.0
	3	37	0.0	5	0.0
	5	38	0.0	7	0.0
	7	18	0.0	6	0.0
	8	87	0.0	15	0.0
	9	42	0.0	0	–
	10	5	0.0	0	–
	11	93	0.0	4	0.0
	14	33	0.0	17	0.0
	15	28	0.0	0	–
	16	43	7.0	0	–
	17	54	0.0	6	0.0
	18	27	0.0	11	0.0
	19	41	0.0	2	0.0
	20	28	0.0	10	0.0
	23	51	0.0	8	0.0
	24	50	0.0	0	–
	25	35	0.0	13	0.0
	26	6	0.0	0	–
	27	14	0.0	11	0.0
	28	41	0.0	2	0.0
	29	30	0.0	2	0.0
	30	38	0.0	5	0.0
	31	16	0.0	0	–
	32	33	0.0	9	0.0
	33	82	0.0	10	0.0
	36	21	0.0	10	0.0
	37	66	0.0	3	0.0
	39	37	0.0	9	0.0
	40	14	0.0	7	0.0
	41	29	0.0	1	0.0
	42	40	0.0	2	0.0
	43	30	0.0	13	0.0
	44	15	0.0	4	0.0
	45	37	0.0	0	–
	46	42	0.0	0	–
	47	24	0.0	17	0.0
	49	15	0.0	2	0.0
	51	28	0.0	1	0.0
	52	82	0.0	2	0.0
2011	1	12	0.0	0	–
	2	32	0.0	2	0.0
	3	39	0.0	3	0.0
	4	44	0.0	0	–
	6	17	0.0	2	0.0
	7	69	0.0	2	0.0
	8	28	0.0	0	–
	10	46	0.0	11	0.0
	11	43	0.0	0	–
	12	34	0.0	2	0.0
	14	42	0.0	8	0.0
	15	41	2.4	3	0.0
	16	37	0.0	0	–
	18	16	0.0	0	–
	19	48	0.0	0	–
	20	85	0.0	0	–
